# Frequently asked questions regarding treatment of Kawasaki disease

**DOI:** 10.21542/gcsp.2017.30

**Published:** 2017-10-31

**Authors:** Jane C. Burns

**Affiliations:** Dept of Pediatrics, University of California San Diego and Rady Children’s Hospital, San Diego, CA, USA

## Introduction

The mainstay of therapy for acute Kawasaki disease (KD) is intravenous immunoglobulin (IVIG), which was first described in a case series from Japan and later proven through a nationwide clinical trial in the U.S. published in 1986^1,2^. Since completion of the initial clinical trials, many questions have arisen regarding the nuances of KD treatment. In the absence of an evidence base, what follows is an attempt to devise rational responses to these questions that draw upon common sense and the personal experience of this author.

## Question 1: Should KD patients be treated if they are diagnosed beyond the tenth day of fever?

**Answer:** Yes, with qualifiers! It is important to understand the source of the “magic” 10-day limit for IVIG treatment. Essentially, the 10-day limit was established for the purpose of conducting the first clinical trial in the US. Jane Newburger and I debated different cut-points for the design of the clinical trial. We needed to establish patient eligibility for entry into the trial and we considered the following issues.

First, we wanted to be able to enroll the maximum number of subjects by liberalizing the number of days of fever. Secondly, we wanted to restrict enrollment to patients who had not already developed coronary artery abnormalities (CAA) as that was our primary endpoint for the trial. We considered a cut-point for enrollment of seven days, but rejected that as too limited because in that era many KD patients were not diagnosed until the end of the first week of fever. We considered a cut-point of 12 days, but rejected that because of the increased risk of enrolling patients who already had CAA and who therefore would not contribute to our primary outcome measure, prevention of CAA. Therefore, 10 days or less of fever was established as our cut-point for enrollment as a balance between efficiency and efficacy.

Returning to basic principles, the goal of IVIG treatment in acute KD is to rapidly reduce inflammation and prevent or halt progression of damage to the coronary arteries. Therefore, it can be reasoned that if a patient is diagnosed beyond the 10th day of fever but still has signs of persistent inflammation (e.g., elevated erythrocyte sedimentation rate (ESR)), that patient should receive IVIG. The ESR may remain elevated in untreated KD patients for weeks beyond the acute febrile phase of KD. The C-reactive protein, however, normalizes even without treatment by the end of the second week. This is consistent with the dynamics of the acute phase response in any self-limited, inflammatory condition.

If the patient is diagnosed late in the course of the illness when the ESR has already fallen below 30 mm/hour and the echocardiogram is normal, then IVIG may not be indicated. However, in the setting of coronary artery dilation or frank CAA, IVIG should always be administered in an attempt to reduce tissue level inflammation regardless of the Illness day (first day of fever = Illness day 1) and systemic markers of inflammation.

With respect to aspirin therapy, patients who present beyond the 10th day of fever who have an elevated platelet count (>450,000/ul) should be treated with 3-5 mg/kg/day of aspirin to inhibit platelet aggregation. Whether or not to use anti-inflammatory doses of aspirin in conjunction with IVIG in these patients with a delayed diagnosis is addressed below.

## Question 2: Should IVIG treatment be delayed until the fifth day of fever?

**Answer:** No. It should be remembered that the “5 days of fever” rule to establish the diagnosis of KD was part of the epidemiologic case definition created by Dr. Kawasaki and a panel of epidemiologists who performed the first Japanese nationwide survey in 1970^3^. The requirement for five days of fever was an attempt to eliminate confusion with most viral rash/fever illnesses, which generally have a shorter duration of fever. Therefore, the “5 day rule” was never intended to guide therapy of the disease, nor was it intended to restrict the diagnosis of KD in cases for which the clinical criteria were already met. In the words of the AHA guidelines, “the diagnosis of KD can be made before the 4th day of fever in the hands of experienced clinicians”^4^.

With respect to the timing of IVIG treatment, an epidemiologic study of KD in Japan published in 2008 has been widely misinterpreted^5^. In this report of 15,940 KD patients in Japan during 2003–2004, 6330 patients received 2 g/kg single infusion IVIG within 10 days of illness onset and 20.3% were classified as IVIG-resistant. Risk factors for IVIG resistance included male sex [odds ratio (OR), 1.21, 95% confidence interval (CI), 1.06–1.37] and receipt of the initial IVIG before the fifth day of illness (OR: 1.89, 95% CI: 1.66–2.15). The authors also noted that IVIG-resistant patients had a significantly higher risk for coronary artery aneurysms (OR: 10.38, 95% CI: 6.98–15.45). The authors concluded that these patients might benefit from administration of adjunctive therapy early during the illness along with the initial IVIG treatment. The study has been widely misinterpreted to mean that somehow giving IVIG before the 5th day of fever was less effective and that IVIG treatment should be withheld until five days of fever had elapsed. Nothing could be farther from the truth! This is an example of “confounding by indication”. In other words, patients who were diagnosed before the fifth day of Illness were likely to be the sickest patients with full criteria and dramatic signs of inflammation on laboratory testing and therefore the clinician felt comfortable making the diagnosis early in the course of the illness. It also follows that the most severely inflamed patients are those most likely to be IVIG resistant. Therefore, treatment should never be withheld and should be administered as soon in the course of the KD as possible.

## Question 3: What dose of aspirin should be used?

**Answer:** It depends! Aspirin has very different pharmacologic effects at different doses, which can be divided into anti-platelet doses (3–5 mg/kg/day), anti-pyretic doses (30–50 mg/kg/day), and anti-inflammatory doses (80–100 mg/kg/day). The initial therapy for KD in the 1970s was high dose aspirin, which was 30–50 mg/kg/day in Asia and 80–100 mg/kg/day in North America. The dose was borrowed from systemic onset juvenile idiopathic arthritis and rheumatic fever. The lower dosing in Asia was due to concern for altered metabolism of the drug due to genetic polymorphisms prevalent in Asian populations. When the first NIH-sponsored clinical trial of IVIG was being designed, the FDA required that the trial be conducted as a randomized comparison of high dose aspirin alone vs. high dose aspirin plus IVIG. This was due to the perception that high dose aspirin was the standard of care, even though trials in Japan had established that aspirin therapy did not reduce the incidence of CAA^6^. Therefore, the original IVIG trial in the U.S. was designed with high-dose aspirin (80-100 mg/kg/day) through the 14th day of Illness in both treatment arms^2^. Unfortunately, once the trial was successfully completed, we were obligated to use high dose aspirin in the treatment of acute KD if we wanted to be consistent with the evidence base.

Over time, meta-analyses and small trials supported the idea that aspirin dose was not critical for IVIG to achieve its beneficial effect^7,8^. Reports also documented significant toxicity associated with high dose aspirin in this patient population^9–11^. Current guidelines from Japan advocate the use of anti-pyretic doses of aspirin (30–50 mg/kg/day) in the acute stage until fever resolves followed by low-dose aspirin (3–5 mg/kg/day) for the anti-platelet effect until markers of inflammation have returned to normal and the echocardiogram shows no changes in the coronary arteries.

When choosing the dose of aspirin in the acute phase of KD, it is important to keep in mind the following issues. First, there is no comparative trial data for aspirin at any dose in acute KD. Second, despite the use of different acute aspirin doses in the US (80–100 mg/kg/day) and Japan (30–50 mg/kg/day) in the IVIG era, the rates of aneurysms are essentially the same between the two countries^12^. Finally, non-steroidal anti-inflammatory drugs and acetaminophen were not available in the early days of KD and thus have never been systematically tested for treatment of the acute phase of the illness. Given the issues of potential toxicity and the lack of evidence that aspirin modifies coronary artery outcome, it would seem reasonable to treat with aspirin at the dose used in Asia and Western Europe (30–50 mg/kg/day) followed by low-dose aspirin until the markers of systemic inflammation have resolved and the echocardiogram has returned to normal.

## Question 4: What therapy should be given in the setting of IVIG resistance, defined as persistent or recrudescent fever 36 h following the end of the initial IVIG infusion?

**Answer:** In patients with recrudescent fever after IVIG treatment, one must first revisit the differential diagnosis of KD to confirm that the correct diagnosis has been made. Next, the patient should be evaluated for a newly acquired infection unrelated to KD as a cause for the new fever. Once these steps have been taken and the diagnosis of KD confirmed, additional anti-inflammatory therapy should be given, as this subset of patients is at highest risk for coronary artery damage^13^. Options for adjunctive therapy include corticosteroids, infliximab, a second dose of IVIG, and cyclosporine^14–17^. However, none of the clinical trials that were designed to answer the question of optimal therapy were adequately powered. A comparative effectiveness trial that compares a second infusion of IVIG to a single dose of infliximab (10 mg/kg) is in progress and is designed to help answer this question (NCT 3065244 aka KIDCARE).

## Question 5: In which patients should early adjunctive therapy be considered?

**Answer:** Patients at highest risk for CAA may benefit from adjunctive therapy, although there are no clinical trial data to inform the decision regarding choice of therapy or dose. This subset can often be identified by having coronary artery dilation on the initial echocardiogram^18^. In a study of 210 consecutive KD patients, 57 patients had CAA. Of these, 46 (81%) had CA dilation or aneurysm on their initial echocardiogram. Thus, the echocardiogram is an effective tool to identify KD patients who may benefit from additional anti-inflammatory therapy. Treatment options are the same as those for IVIG resistance.

An additional subset of KD patients who may benefit from adjunctive therapy is infants less than 6 months of age. In a study of 88 infants ≤ 6 months of age, a larger proportion had dilated or aneurysmal coronary arteries on the initial echocardiogram as compared to those ≥ 6 months of age (43.4% vs. 19.5%)^19^. Therefore, these young patients represent a vulnerable population for whom intensification of initial therapy may be appropriate.

Future therapy of acute KD will likely include some system of risk stratification and intensification of anti-inflammatory therapy for those at greatest risk. Clinical trial data are needed to clarify the role of adjunctive therapies including steroids, infliximab, cyclosporine, and anakinra to achieve the best outcome for our patients^20^.
